# Clinical researchers’ insights on key data for eligibility screening in clinical studies

**DOI:** 10.1017/cts.2024.617

**Published:** 2024-10-16

**Authors:** Betina Idnay, Emily R. Gordon, Aubrey S. Johnson, Jordan G. Nestor, Karen Marder, Chunhua Weng

**Affiliations:** 1 Department of Biomedical Informatics, Columbia University, New York, NY, USA; 2 Vagelos College of Physicians and Surgeons, Columbia University, New York, NY, USA; 3 Department of Neurology, Columbia University, New York, NY, USA; 4 Department of Medicine, Columbia University, New York, NY, USA

**Keywords:** Informatics, clinical research, eligibility prescreening, freelisting, clinical research staff

## Abstract

**Introduction:**

Clinical research is critical for healthcare advancement, but participant recruitment remains challenging. Clinical research professionals (CRPs; e.g., clinical research coordinator, research assistant) perform eligibility prescreening, ensuring adherence to study criteria while upholding scientific and ethical standards. This study investigates the key information CRP prioritizes during eligibility prescreening, providing insights to optimize data standardization, and recruitment approaches.

**Methods:**

We conducted a freelisting survey targeting 150 CRPs from diverse domains (i.e., neurological disorders, rare diseases, and other diseases) where they listed essential information they look for from medical records, participant/caregiver inquiries, and discussions with principal investigators to determine a potential participant’s research eligibility. We calculated the salience scores of listed items using Anthropac, followed by a two-level analytic procedure to classify and thematically categorize the data.

**Results:**

The majority of participants were female (81%), identified as White (44%) and as non-Hispanic (64.5%). The first-level analysis universally emphasized age, medication list, and medical history across all domains. The second-level analysis illuminated domain-specific approaches in information retrieval: for instance, history of present illness was notably significant in neurological disorders during participant and principal investigator inquiries, while research participation was distinctly salient in potential participant inquiries within the rare disease domain.

**Conclusion:**

This study unveils the intricacies of eligibility prescreening, with both universal and domain-specific methods observed. Variations in data use across domains suggest the need for tailored prescreening in clinical research. Incorporating these insights into CRP training and refining prescreening tools, combined with an ethical, participant-focused approach, can advance eligibility prescreening practices.

## Introduction

Clinical research underpins medical advancements, healthcare policies, and patient outcomes. However, recruiting eligible participants remains a significant challenge, often causing delays, additional costs, and reduced data quality due to inadequate sample diversity and size [1–3]. Clinical research professionals (CRPs), including coordinators and research assistants, are crucial conduits between investigators and potential participants [4,5]. They are often the first interaction point for prospective participants, significantly influencing recruitment success, participant diversity, enrollment numbers, and the participants’ understanding and willingness to participate [6,7]. CRPs employ various strategies to address recruitment challenges, including social media outreach, community programs, database optimization, and coordination with clinical teams within healthcare institutions [8–12]. Each method has advantages and limitations, demanding a nuanced approach to specific research conditions.

A critical aspect of the CRP role is eligibility prescreening, where the CRP reviews medical records, contacts patients and caregivers, and collaborates with the principal investigator to assess eligibility before informed consent [13]. This phase of the recruitment process aims to ensure potential participants are likely to fulfill the study’s criteria before any informed consent is sought or detailed medical record review is conducted [14]. Its impact extends beyond mere eligibility; it fundamentally affects the scientific integrity, generalizability, and ethical implications associated with the research. Inadequate prescreening can limit access to potential participants or cause unnecessary distress during consent and screening upon discovering ineligibility, which could have been avoided [13], thereby skewing research outcomes and raising ethical concerns [15].

Faced with these multifaceted challenges, our study addresses the challenges of eligibility prescreening by exploring where CRPs obtain necessary information and what patient information they consider crucial. We aim to streamline prescreening procedures to enhance recruitment for high-quality clinical research. We conducted a comprehensive survey targeting CRPs across various roles and domains to understand their real-world practices and preferences in eligibility prescreening. The insights from this survey will inform the development of advanced prescreening tools and specialized training programs for CRPs. This effort aims to create a more efficient, effective, and ethically grounded clinical research environment, benefiting the medical research community and diverse patient populations.

## Methods

### Study design and sampling

This study was approved by the Columbia University Institutional Review Board (#AAAD1873). We conducted a prospective online freelisting survey via Qualtrics (Provo, UT) with 150 CRPs from various institutions within and beyond the Columbia University network recruited through institutional listservs and online community forums using stratified, convenience, and snowball sampling to ensure diverse perspectives. Participants were at least 18 years old with CRP experience within the last 12 months and involved in identifying potential participants for recruitment. We used purposive sampling to target three CRP subgroups: neurological disorders research (*n* = 50), rare diseases research (*n* = 50), and other disease domains (*n* = 50). Neurological disorders research was chosen for its diverse conditions (i.e., from more prevalent disorders such as stroke to less common conditions such as frontotemporal dementia) and unique recruitment challenges (e.g., capacity to consent), rare diseases research for its sourcing complexities from limited populations, and other disease domains for broad representation of clinical research contexts.

Freelisting is a qualitative research technique focused on elucidating the spectrum and organizing principles of terms, concepts, or items that a specific community associates with a given subject matter [16,17]. Participants are asked to list as many items, terms, or concepts related to a designated topic in response to an open-ended question to allow unrestricted listing, providing insights into the collective cognitive structure of the participants [16,18]. This method captures the richness and diversity of perceptions, offering a multifaceted view of complex subjects. In healthcare research, freelisting identified terminologies related to symptoms, health beliefs, and cultural perspectives on treatment [19–23]. Our study aimed to understand the terms, concepts, and information CRPs consider essential during the eligibility prescreening for participant recruitment.

### Data collection

We conducted a 20-minute online freelisting survey (see Supplementary File A) to understand what CRPs consider crucial in evaluating patient eligibility for clinical research. The survey aimed to identify the main sources of information CRPs consult, the patient-specific details they prioritize, and the role of dialogue between CRPs and principal investigators and potential research participants in assessing eligibility. Participants listed information consulted in medical records, inquiries made directly to patients or caregivers, and discussions with principal investigators. A demographic questionnaire was included to identify trends or biases. Participants received a $40 Amazon gift card upon completion.

### Data analysis

Descriptive statistics summarized the survey data. We calculated the salience score for each listed item using Anthropac (Lexington, KY) version 4.98, a tool designed to evaluate data from lists. The salience score represents the weighted average of an item’s (inverse) rank across multiple lists, with each list weighted by its number of items [16,18]. More specifically, the lower an item’s rank on a list (indicating its higher importance), the higher its salience score. Each list contributes to the score in proportion to its total number of items, thereby giving weight to the prevalence of an item in a broader context. The salience score captures an item’s frequency and prominence, providing insights into the perceived importance among respondents.

Responses were stratified into the three primary disease domains: neurological disorders, rare diseases, and other unspecified domains. Information was categorized based on three sources: caregiver or potential participant inquiry, medical records review, and principal investigator inquiry. Four coders, including one primary coder (BI) and three others (JGN, ERG, and ASJ), with domain-specific recruitment experience, classified synonyms and categories. Regular meetings ensured consensus on discrepancies.

This analysis had two sequential levels: first, identifying and categorizing synonyms to consolidate the list and ensure accurate representation of unique concepts; second, grouping categorized synonyms into broader thematic categories. This two-tiered approach provided granular insights and broader patterns, offering a comprehensive understanding of what CRPs consider essential during the eligibility prescreening phase of participant recruitment.

## Results

### Participant characteristics

The study included 150 CRPs from diverse disease domains, including neurological disorders, rare diseases, and other areas. Table 1 shows participant demographics: average age was 30.4 years (SD: 7.8; range: 20–60), 81% were female, 44% identified as White or Caucasian, 25% as Asian or Asian American, and 12% as Black or African American. Majority were non-Hispanic (64.5%). Half had a bachelor’s degree, and about one-third had a master’s degree. Predominant roles were clinical research coordinators (57%) and research assistants (20%). Experience in clinical research varied, with about a quarter having one to less than two years or two to less than five years of experience.

**Table 1.** Participant characteristics (*N* = 150)

Characteristic	Neurological disorders (*n* = 50)	Rare disease (*n* = 50)	Other (*n* = 50)	Total
Age in years, mean (SD)[range]	31.7 (9.9) [22-60]	29.7 (7.2) [20-60]	31.1 (8.5) [22-59]	30.4 (7.8) [20-60]
Race, *n* (%)				
American Indian or Alaskan Native	–	1 (2)	–	1 (1)
Asian or Asian American	9 (18)	13 (26)	15 (30)	37 (25)
Black or African American	10 (20)	3 (6)	5 (10)	18 (12)
Native Hawaiian/Other Pacific Islander	–	–	1 (2)	1 (1)
White or Caucasian	20 (40)	25 (50)	21 (42)	66 (44)
Multiracial	5 (10)	5 (10)	5 (10)	15 (10)
Something else (please specify)	1 (2)	3 (6)	2 (4)	6 (4)
Prefer not to answer	5 (10)	–	1 (2)	6 (4)
Ethnicity, *n* (%)				
Non-Hispanic	32 (64)	31 (62)	34 (68)	97 (645)
Hispanic	16 (32)	19 (38)	16 (32)	51 (34)
Prefer not to answer	2 (4)	–	–	2 (1)
Gender, *n* (%)				
Female	40 (80)	41 (82)	41 (82)	122 (81)
Male	8 (16)	8 (16)	9 (18)	25 (17)
Gender variant/non-conforming	1 (2)	–	–	1 (1)
Prefer not to answer	1 (2)	1 (2)	–	2 (1)
Level of education, *n* (%)				
Some college	–	3 (6)	–	3 (2)
Associate degree	–	1 (2)	3 (6)	4 (3)
Bachelor”s degree	29 (58)	21 (42)	25 (50)	75 (50)
Master”s degree	16 (32)	18 (36)	16 (32)	50 (33)
Doctoral degree	5 (10)	7 (14)	6 (12)	18 (12)
Research position, *n* (%)				
Clinical research coordinator	22 (44)	31 (62)	32 (64)	85 (57)
Genetic counselor	–	2 (4)	–	2 (1)
Investigator	–	1 (2)	–	1 (1)
Postdoctoral research scientist	–	–	1 (2)	1 (1)
Research administrator	1 (2)	1 (2)	1 (2)	3 (2)
Research assistant	16 (32)	7 (14)	7 (14)	30 (20)
Clinical research nurse	–	1 (2)	2 (4)	3 (2)
Research/Project/Program manager	8 (16)	4 (8)	6 (12)	18 (12)
Other	3 (6)	3 (6)	1 (2)	7 (5)
Years as clinical research professional (%), *n* (%)				
less than a year	10 (20)	8 (16)	12 (24)	30 (20)
1 year to less than 2 years	12 (24)	12 (24)	12 (24)	36 (24)
2 years to less than 5 years	12 (24)	16 (32)	14 (28)	42 (28)
5 years to less than 10 years	7 (14)	11 (22)	6 (12)	24 (16)
10 years or over	9 (18)	3 (6)	6 (12)	18 (12)
Top 3 most common disease categories	Alzheimer’s disease and related dementias Cerebrovascular disorders Movement disorders	Cystic Fibrosis Thrombocytopenia and related disorders Lupus and Lupus-related disorders	Cardiovascular disease Cancer Infectious diseases	

*Synonyms and Categories Identified During Potential Participant Inquiry*.

Supplementary Table S1 details categories and synonyms. In neurological disorders, 15 categories were identified, with “History of present illness” being the most prevalent, including cognitive status to disease onset age. “Research participation” highlighted aspects of readiness for research. The rare diseases domain revealed 14 categories, some overlapping with neurological disorders, with unique specifics like the last menstrual period in “Obstetric and gynecological history” and Raynaud’s syndrome in “Past medical history.” Other disease domains had 17 categories. “History of present illness” covered aspects like symptom duration, while unique categories like “Physical activity status” examined activity level. “Social History” emphasized factors like incarceration, highlighting various social elements affecting health or research eligibility.

### Synonyms and categories identified during medical records review

In neurological disorders, 16 categories were identified (Supplementary Table S2). “Clinical care history” included healthcare provider and hospitalization, while “Cognitive test results” comprised Mini-Mental Status Examination. “Personal information” included information such as name, and “Research Eligibility” and “Research participation” spotlighted research adherence, and family willingness, respectively. The rare disease domain listed 18 categories, overlapping with neurological disorders in “Clinical care history” and “History of present illness,” but with unique terms including “lung infection” and “Mortality.” Other disease domains had 17 categories, with “Diagnostic results” covering genetic results, and “Obstetric and gynecological history” including conception method. “Research eligibility” covered adherence to treatment regimens.

### Synonyms and categories identified during principal investigator inquiry

In neurological diseases, 17 categories were identified (Supplementary Table S3), covering clinical care to research aspects. “Clinical care history” included terms like healthcare provider, while “Research participation” covered study logistics and patient willingness. Rare diseases had 14 categories, with unique terms like fetal abnormalities in “Obstetric and gynecological history.” “Harmful substance use” covered alcohol and smoking histories, highlighting lifestyle impacts on disease. Other disease domains showcased 16 categories, with diverse “History of present illness,” focusing on diagnosis to symptom duration, “Physical activity status” on mobility, and a new “Recruitment” category reflected patient engagement procedures.

### Similarities and differences in information gathering by disease domain

For neurological diseases, respondents used similar information sources (i.e., potential research participant, medical records, and principal investigator) to inquire about clinical care history, clinical documentation, demographics, diagnostic results, family history, history of present illness, past medical history, personal information, research eligibility, research participation, treatment or medication, and visit data. Cognitive test results were reviewed through medical records review and principal investigator inquiries, delving into mental status, mood, and prognosis.

For rare diseases, demographics, diagnostic results, history of present illness, obstetric and gynecological history, past medical history, personal information, research eligibility, research participation, treatment or medication, and visit data are consistent across all sources. Clinical care history was only inquired about from the medical records and potential participant inquiries. Physical function and harmful substance use were comprehensively examined across all data sources.

Regarding other diseases, common categories across all stages were clinical care history, demographics, diagnostic results, history of present illness, obstetric and gynecological history, past medical history, personal information, research eligibility, research participation, harmful substance use, treatment or medication, and visit data. The principal investigator inquiry included nuances like surgical candidacy and the need for dental screenings or glucose management, which were less prominent in other domains.

Core categories were consistent across stages and domains, but the depth and specificity of inquiries varied. The participant inquiry focused on broader categories, the medical records review provided detailed clinical history, and the principal investigator inquiry delved deeper into aspects critical for research participation and the disease.

### First-level analysis: synonyms by disease domain

The first level of analysis categorized items by synonyms, revealing distinct patterns of information prioritization among CRPs across three disease domains (Table 2). For detailed salience scores and items, see Supplementary Tables S4–S6. “Age” was the most salient item in neurological disease during medical records reviews (0.690). “Gender” and “Age” topped potential participant inquiries and principal investigator discussions, scoring 0.204 and 0.193, respectively. “Medication list” and “Medical history” were also emphasized across all categories.

**Table 2.** Top 10 synonyms by disease domain: a first-level analysis of salience scores

	Potential participant inquiry			Medical records review			Principal investigator inquiry		
	Item	*n*	Salience score	Item	*n*	Salience score	Item	*n*	Salience score
	**NEUROLOGICAL DISORDERS DOMAIN**								
1	Gender	10	0.204	Age	34	0.690	Age	10	0.193
2	Medication list	18	0.194	Diagnosis	20	0.283	Past medical history	11	0.139
3	Past medical history	11	0.162	Medication list	19	0.180	Medication list	13	0.138
4	Ability to attend study visits	8	0.134	Residence location	13	0.152	Diagnosis	9	0.118
5	Willingness to participate in research	12	0.103	Past medical history	14	0.135	Laboratory results	6	0.099
6	Diagnosis	8	0.095	Comorbidities	9	0.103	Principal investigator”s eligibility determination	6	0.091
7	Family history	6	0.093	Family history	6	0.086	Protocol eligibility criteria	5	0.088
8	Symptoms	9	0.087	Date of birth	4	0.074	Family history	6	0.065
9	Capacity to consent	5	0.071	Language	7	0.073	Imaging results	6	0.061
10	Participant concerns about research	4	0.063	Research participation history	8	0.072	Demographics	3	0.061
	**RARE DISEASE DOMAIN**								
1	Availability to attend study visit	12	0.205	Age	27	0.534	Medication list	12	0.192
2	Age	10	0.180	Medication list	16	0.177	Medical history	12	0.175
3	Interest in research participation	13	0.171	Laboratory results	17	0.157	Age	7	0.152
4	Medication list	13	0.170	Genetic testing result	12	0.156	Principal investigator”s eligibility determination	9	0.140
5	Medical history	12	0.110	Diagnosis	13	0.143	Laboratory results	7	0.100
6	Fetal abnormalities	4	0.083	Imaging results	7	0.101	Diagnosis	6	0.093
7	Diagnosis	5	0.071	Fetal abnormalities	6	0.099	Protocol eligibility criteria	5	0.083
8	Ability to perform research procedures	4	0.069	Pulmonary function test	8	0.086	Pulmonary function test	5	0.082
9	Disease status	5	0.066	Date of birth	4	0.080	Disease status	6	0.071
10	Current treatment/intervention	4	0.061	Medical history	8	0.077	Genetic testing result	4	0.071
	**OTHER DISEASE DOMAIN**								
1	Age	12	0.247	Age	39	0.763	Age	15	0.304
2	Availability to attend study visit	12	0.204	Medication list	20	0.230	Medication list	20	0.242
3	Medication list	15	0.197	Laboratory results	16	0.224	Protocol eligibility criteria	11	0.168
4	Past medical history	12	0.140	Comorbidities	18	0.220	Comorbidities	9	0.147
5	Capacity to consent	11	0.136	Diagnosis	9	0.132	Past medical history	7	0.111
6	Ability to perform research procedures	5	0.090	Past medical history	12	0.118	Laboratory results	9	0.110
7	Comorbidities	7	0.087	Imaging results	8	0.078	Adherence	5	0.083
8	Willingness to participate in research	7	0.081	Past surgical history	9	0.072	Imaging results	6	0.082
9	Interest in research participation	4	0.065	Gender	5	0.068	Diagnosis	5	0.074
10	Healthcare provider	5	0.060	Disease status	6	0.067	Principal investigator”s eligibility determination	5	0.066

*Second-Level Analysis: Categorization by Diseases Domain*.

In rare diseases, “Availability to attend study visit” was most salient in potential participant inquiries (0.205), followed by “Age” (0.180) and “Interest in research participation” (0.171). “Age” was prioritized in medical records reviews (0.534), followed by “Medication list” (0.177) and “Laboratory results” (0.157). Principal investigator inquiries focused on the “Medication list” (0.192), “Medical history” (0.175), and “Age” (0.152).

For other diseases, potential participant inquiries highlighted “Age” (0.247), “Availability to attend study visit” (0.204), and “Medication list” (0.197). Medical records reviews were dominated by “Age” (0.763), succeeded by “Medication list” (0.230) and “Laboratory results” (0.224). Principal investigator inquiries emphasized “Age” (0.304), “Medication list” (0.242), and “Protocol eligibility criteria” (0.168).

**Figure 1.**
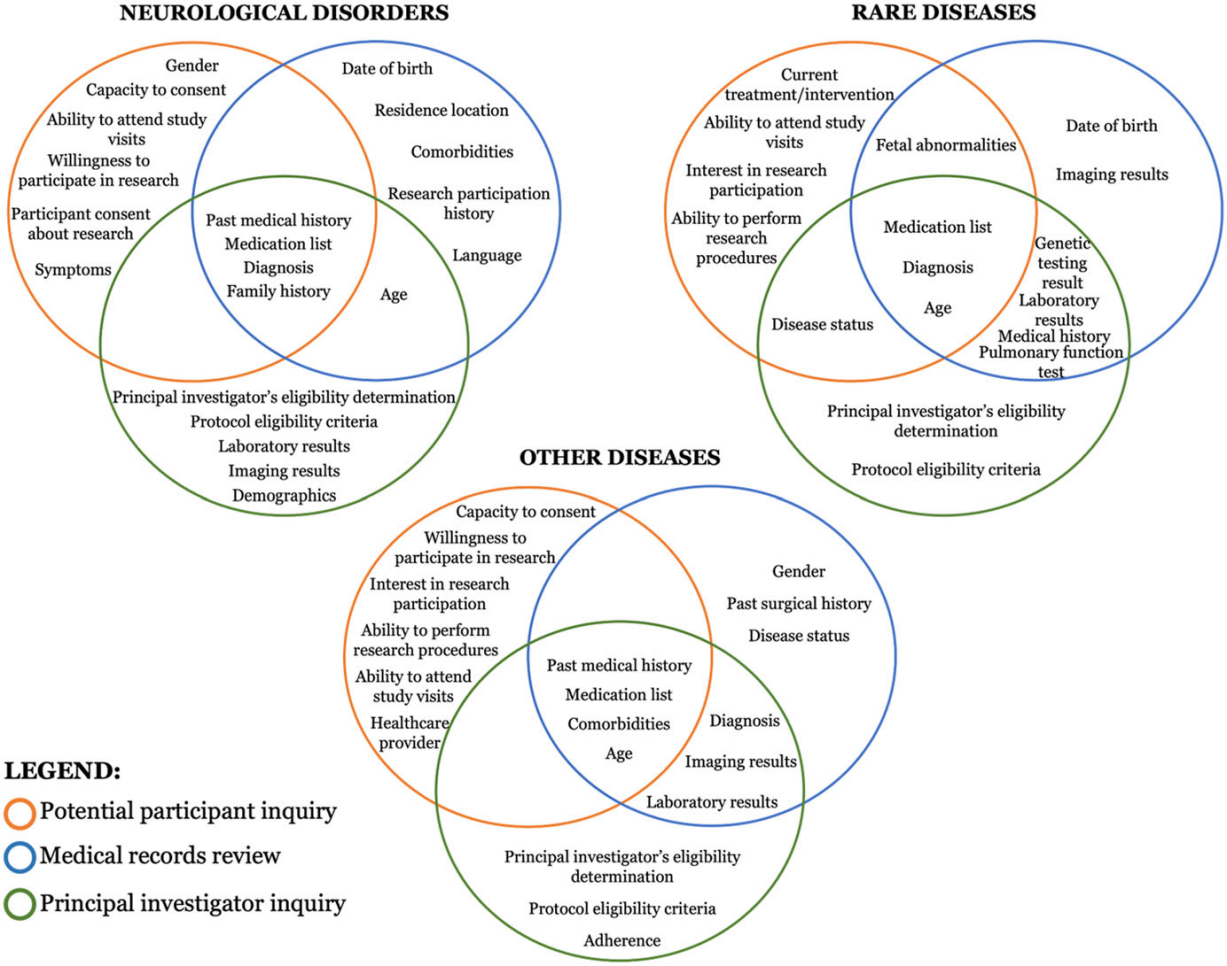
Shared and divergent information inquiry from data sources across domains.

A key similarity across all domains (Figure 1) was the high importance of “Age” and the commonality of “Medication List.” “Past Medical History” and “Diagnosis” also consistently appearing, albeit at varying ranks. Differences were evident in the emphasis on specific factors within each disease domain. In the neurological disorders, potential participant inquiries highlighted “Ability to attend study visits” and “Willingness to participate in research,” with medical records review often including “Residence location” and “Comorbidities.”

In rare diseases, potential participant inquiries and medical records reviews uniquely highlighted “Fetal abnormalities.” The medical records review and the principal investigator’s inquiry mentioned “Genetic testing result” and “Pulmonary function test.” This domain did not saliently inquire about “Past medical history” from potential participants, nor did “Family history” rank in the top ten most salient terms, but “Genetic testing results” were emphasized.

For other diseases, potential participant inquiry prioritized “Availability to attend study visit,” while medical records review focused on “Comorbidities” and “Past surgical history.” Principal investigator inquiries in this domain distinctively featured “Adherence.”

Overall, these variations revealed each inquiry method’s differing perspectives and priorities. Potential participant inquiries focused on participation feasibility and willingness factors, medical record reviews emphasized historical and diagnostic data, and principal investigator inquiries balanced logistical aspects like “Medication list” and specific medical details like “Laboratory results” and “Diagnosis.”

In our second-level analysis, detailed in Table 3, we explored the frequency and salience of categories within three disease domains. In neurological disorders, “History of present illness” was significant in potential participant and principal investigator inquiries, with salience scores of 0.394 and 0.334, respectively. Interestingly, “Diagnostic results” were exclusively reviewed from the medical records and inquired from the principal investigator but were not asked from potential participants. “Harmful substance use” was examined only from potential participants. Demographic information was primarily sought during medical records reviews, with a salience score of 0.706.

**Table 3.** Categories by disease domain: a second-level analysis of salience scores

	Potential participant inquiry			Medical records review			Principal investigator inquiry		
	Item	*n*	Salience score	Item	*n*	Salience score	Item	*n*	Salience score
	**NEUROLOGICAL DISORDERS DOMAIN**								
1	History of present illness	29	0.394	Demographics	37	0.706	History of present illness	24	0.334
2	Research participation	23	0.295	History of present illness	31	0.413	Research eligibility	19	0.29
3	Demographics	16	0.289	Past medical history	27	0.297	Demographics	14	0.269
4	Past medical history	16	0.232	Personal information	18	0.228	Past medical history	18	0.218
5	Treatment or medication	19	0.194	Treatment or medication	20	0.178	Research participation	14	0.186
6	Research eligibility	11	0.175	Diagnostic results	15	0.173	Treatment or medication	17	0.174
7	Personal information	9	0.096	Research participation	10	0.103	Diagnostic results	11	0.151
8	Family history	6	0.092	Clinical care history	7	0.094	Functional status	4	0.069
9	Functional status	5	0.085	Family history	6	0.086	Personal information	5	0.067
10	Harmful substance use	3	0.048	Functional status	6	0.071	Family history	6	0.067
11	Past surgical history	7	0.047	Clinical documentation	4	0.055	Clinical care history	4	0.044
12	Social history	3	0.046	Research eligibility	6	0.054	None	2	0.041
13	Clinical care history	2	0.026	Past surgical history	8	0.046	Visit data	3	0.032
14	Clinical documentation	1	0.010	Visit data	2	0.030	Past surgical history	2	0.031
15	Visit data	1	0.010	Cognitive test results	5	0.026	Social history	4	0.025
16	–	–	–	Social history	2	0.016	Cognitive test results	2	0.023
17	–	–	–	None	1	0.004	Clinical documentation	1	0.010
	**RARE DISEASE DOMAIN**								
1	Research participation	29	0.447	Demographics	31	0.574	Past medical history	17	0.268
2	Treatment or medication	20	0.270	Diagnostic results	32	0.382	Diagnostic results	16	0.250
3	Past medical history	20	0.252	History of present illness	25	0.284	Research participation	17	0.242
4	History of present illness	15	0.246	Treatment or medication	22	0.218	History of present illness	17	0.230
5	Demographics	11	0.198	Past medical history	18	0.213	Treatment or medication	15	0.223
6	OBGYN history	9	0.137	OBGYN history	10	0.136	Research eligibility	11	0.182
7	Research eligibility	8	0.120	Personal information	8	0.130	Demographics	7	0.152
8	Diagnostic results	8	0.118	Clinical care history	6	0.100	OBGYN history	5	0.068
9	Personal information	5	0.074	Clinical documentation	6	0.063	Personal information	5	0.065
10	Clinical documentation	3	0.033	Past surgical history	10	0.061	Physical function	2	0.036
11	Clinical care history	4	0.030	Visit data	5	0.055	Visit data	2	0.025
12	Harmful substance use	4	0.018	Physical function	4	0.053	Harmful substance use	2	0.020
13	Past surgical history	3	0.013	Family history	2	0.033	Past surgical history	1	0.014
14	Family history	1	0.010	Harmful substance use	3	0.022	Clinical care history	1	0.007
15	–	–	–	Mortality	1	0.020	–	–	–
16	–	–	–	Research participation	2	0.012	–	–	–
17	–	–	–	Recruitment	1	0.007	–	–	–
18	–	–	–	Research eligibility	1	0.005	–	–	–
	**OTHER DISEASE DOMAIN**								
1	Research participation	25	0.429	Demographics	40	0.775	Demographics	17	0.336
2	Demographics	17	0.314	Past medical history	30	0.355	Research eligibility	23	0.334
3	Past medical history	22	0.292	Diagnostic results	21	0.288	Past medical history	17	0.268
4	Treatment or medication	18	0.199	History of present illness	21	0.234	Treatment or medication	24	0.261
5	Research eligibility	14	0.175	Treatment or medication	22	0.227	Diagnostic results	13	0.166
6	History of present illness	9	0.106	Visit data	10	0.096	History of present illness	12	0.155
7	Clinical care history	8	0.085	Past surgical history	12	0.087	Research participation	6	0.106
8	OBGYN history	7	0.080	Clinical care history	9	0.073	Visit data	5	0.059
9	Harmful substance use	6	0.070	Personal information	6	0.066	Past surgical history	6	0.055
10	Social history	4	0.063	Research participation	5	0.064	Clinical care history	5	0.054
11	Personal information	4	0.057	OBGYN history	7	0.054	Clinical documentation	3	0.049
12	Diagnostic results	4	0.048	Clinical documentation	5	0.047	Recruitment	3	0.048
13	Past surgical history	4	0.041	Research eligibility	5	0.046	OBGYN history	4	0.035
14	Visit data	4	0.040	Harmful substance use	2	0.027	Harmful substance use	2	0.028
15	Family history	2	0.036	Physical activity status	1	0.017	Personal information	2	0.025
16	Other	3	0.032	Family history	1	0.016	Social history	1	0.017
17	Contact information	1	0.021	Social history	1	0.009	Physical activity status	1	0.004
18	Physical activity status	2	0.016	–	–	–	–	–	–

*Note: OBGYN: Obstetrics and gynecology.*

In rare diseases, “Research participation” was most salient in potential participant inquiries (0.447). Demographic information, “Mortality,” and “Recruitment” were primarily sought during medical records reviews. “Family history” and “Clinical documentation” were not discussed with principal investigators.

In other disease domains, “Research participation” and “Demographics” were highly frequent and salient across all aspects of clinical research. “Contact information” was asked only from potential participants, and “Recruitment” was exclusively requested from the principal investigator. “Family history” was notably absent in principal investigator inquiries.

“Demographics” and “History of present illness” were consistently significant across domains. Unique patterns were observed, such as “Diagnostic Results” and “Harmful substance use” in neurological disorders, which are context-specific, providing a nuanced understanding of category relevance in clinical research.

## Discussion

This study used a freelisting survey to investigate CRPs perspectives across neurological disorders, rare diseases, and other disease domains, engaging 150 CRPs to delineate the complexities of eligibility prescreening. This approach enriched the investigation by identifying and prioritizing elements deemed most important by CRPs [16,17]. The results highlighted the diversity and complexity of prescreening procedures across disease domains, emphasizing the need for tailored approaches in clinical research.

First-level analysis revealed that age consistently emerged as crucial information across all domains and inquiry methods, influencing participant selection, treatment approach, and study outcomes. Medication lists were also salient, indicating the significance of understanding participants’ concurrent therapies. Each disease domain showcased unique priorities, with neurological disorders focusing on logistical considerations, participants’ research inclination, and rare diseases prioritizing genetics. This juxtaposition highlights the necessity of domain-specific knowledge and the tailored approach required in clinical research. Inquiry methods varied, with potential participant inquiries focusing on participation feasibility, medical record reviews on historical data, and principal investigator inquiries combining both. This divergence illuminates the multifaceted nature of clinical research and underscores the importance of a holistic approach, integrating both logistical and medical considerations, for optimal research outcomes.

The second-level analysis demonstrates that “History of present illness” was prioritized in neurological disorders, especially in participant and principal investigator inquiries. At the same time, “Diagnostic results” were focal during medical records reviews and principal investigator inquiries. “Harmful substance use” was exclusively inquired during participant interactions. The difference between the specific categories queried and the consistent focus on demographic data in medical records reviews highlights how CRPs prioritize the information they seek depending on the source, using this strategy to make eligibility prescreening more efficient. In rare diseases, “Research participation” was crucial in participant inquiries, while “Mortality” was exclusively explored in medical records reviews. The limited focus on family history and clinical documentation in principal investigator inquiries highlighted the genetic emphasis in rare disease trials. In other disease domains, “Research participation” and “Demographics” were generally salient, with specific inquiries into contact information and recruitment reflecting varied informational demands. Additionally, it is noteworthy that there is a conspicuous absence of attention to race and ethnicity, especially given that many trials now set caps based on these factors [24]. Considering socioeconomic status, education, and an individual’s capacity to participate is equally crucial, reflecting the evolving landscape of trial prerequisites [25]. The specific patient-oriented inquiries into contact information and principal investigator-exclusive discussions around recruitment offer a peek into the varied, stakeholder-specific informational demands and necessitate a reflective consideration of tailored communication strategies.

These findings underscore the multifaceted nature of clinical research and the need for a holistic approach integrating logistical and medical considerations. Based on these insights, we propose thematic recommendations (Table 4) to develop training and frameworks, ensuring robust and specialized recruitment strategies tailored to each domain. These findings also pave the way for a fine-tuned, domain-oriented approach to developing training and frameworks, ensuring that recruitment strategies are universally robust and aptly specialized [26].

**Table 4.** Recommended practices for clinical research eligibility prescreening

Recommendation	Description
Standardization of universal data types	Given the consistent importance of certain variables like “Age,” “Medication list,” and “Medical history” across varied research domains, there should be a standardized framework for collecting, documenting, and analyzing these data types across all clinical documentation. Standardization can enhance the efficiency of the prescreening process and ensure consistency in data interpretation, irrespective of the disease domain.
Domain-specific adaptability	While some data types are universally relevant, each research domain has its unique set of priorities. Clinical research practices should incorporate domain-specific prescreening modules that allow for tailored approaches. For example, the emphasis on genetic testing results in the rare disease domain or the notable relevance of cognitive testing results in neurological disorders should be distinctly addressed in domain-specific modules.
Dynamic eligibility prescreening tools	Reflecting the dynamic nature of clinical research, our findings suggest the need for prescreening tools that can adapt to new information. We propose the development of informatics-driven prescreening tools that update in real-time to accommodate the emerging trends in the research domain. These tools should offer an intuitive interface, allowing CRPs to efficiently navigate between universal and domain-specific prescreening criteria.
Strengthening ethical considerations	Based on our findings, where the majority of CRPs emphasized the significance of patient history and medication lists, we recommend that ethical considerations be interwoven into the fabric of clinical research beyond mere compliance. We advocate for the implementation of comprehensive training for CRPs to enhance their ability to navigate the ethical complexities highlighted by our participants, particularly in relation to recruitment and prescreening. Training should focus on a deep understanding of potential risks, benefits, data privacy, and the importance of upholding participant well-being—core elements identified as crucial by our study participants.
Robust data protection protocols	Reflecting the concerns about data privacy raised in our survey, we urge the establishment of stringent data protection measures. Our data shows that CRPs are acutely aware of the volume of personal and medical data collected, underscoring the need for clear communication about how participant data will be utilized, stored, and protected. Such transparency is not only mandated by regulations but is also a key factor in building trust, as suggested by the feedback from our study’s participants.
Continuous professional development	In alignment with the trends observed in our data, which indicate a need for ongoing education in prescreening practices, we recommend that CRPs should have access to continuous learning opportunities. Workshops, webinars, and specialized training sessions should be designed to keep pace with the evolving landscape, as noted by the participants in our study, ensuring CRPs remain effective in their roles.
Reevaluation and feedback	Our research underscores the evolving nature of clinical research practices, as indicated by CRP feedback. We advocate for the regular re-assessment of prescreening practices, which should be grounded in systematic feedback from the CRPs. Such reevaluations are proposed not as an immediate precursor to practice changes but as a means to identify potential areas for improvement. It is essential that any subsequent modifications to prescreening strategies, informed by this feedback, are subjected to a thorough effectiveness evaluation. This evaluation should measure the impact of changes on the overall recruitment process and outcomes, ensuring that adaptations are not only responsive to the needs highlighted by our study but are also empirically shown to enhance the prescreening process.

*Note:* CRP: clinical research professional.

### Implications for clinical research practices

Given the universal and domain-specific elements in eligibility prescreening, our findings highlight the need for a refined approach to clinical research practices, particularly regarding participant prescreening strategies. The consistent importance of “Age,” “Medication list,” and “Medical history” across research domains posits these as essential starting points. Nevertheless, distinct nuances within each domain require a bifurcated strategy, blending universal principles with adaptability to domain-specific variables.

Extending from Miller et al.’s study [27] on demographics in research participant selection, our findings suggest that while demographic data, such as age and gender, are vital, their salience varies across domains. Acknowledging the overarching and domain-specific components, it becomes imperative to integrate a multifaceted strategy into clinical research practices by reconfiguring existing frameworks and training modules to embody a more nuanced, contextually relevant approach that resonates with the shared cognitive schemas among CRPs and is congruent with the specificities intrinsic to each research domain. The “one-size-fits-all” approach to CRP training risks impeding the nuanced operation required in distinct research domains [5,13]. Specialized training and resources will help CRPs understand and implement domain-specific variations.

Enhancing resources and tools to encapsulate these findings is paramount. Improving eligibility prescreening tools to incorporate generalized prescreening criteria with an adjustable interface for domain-specific variables could offer CRPs a flexible platform [28]. These tools, integrated with informatics capabilities, can evolve in real time, aligning prescreening criteria with emerging trends [29]. Continuous professional development, including workshops and webinars on prescreening practices, will strengthen CRPs’ ability to navigate participant recruitment adeptly and effectively. Embedding these enriched training paradigms and dynamic resources will improve the precision and efficiency of prescreening strategies, fostering continuous learning and adaptability in CRPs’ practice.

### Ethical considerations and participant-centered approach

Our research emphasizes a participant-focused approach in clinical studies, underscoring the importance of respecting each participant’s unique health context. Ethical considerations are central to the prescreening process, merging scientific rigor with a dedication to participant well-being [30]. Beyond determining eligibility, ethical considerations ensure scientific integrity and participant safety. Detailed understanding of participants’ medical histories is essential, both operationally and ethically, to prevent unintentional harm.

This ethical duty involves assessing risks and benefits for participants through a detailed insight into their medical histories to mitigate potential risks preemptively. A participant-centered model incorporates patient perspectives and experiences, meets ethical standards, and reflects participants’ realities. For example, understanding the challenges of attending study visits, as shown in our findings, urges researchers to create feasible and compassionate participant pathways. Consent forms should be crafted at a 5th-grade reading level to ensure accessibility and comprehension [31]. Data privacy and autonomy are crucial, given the personal information collected during prescreening. Stringent data protection measures and clear communication regarding data handling are vital to maintaining ethical standards [32].

### Limitations and future directions

While our study provides invaluable insights, it has limitations. The sample, although diverse, may not capture the full spectrum of CRP roles and experiences. Additionally, the systematic and thorough categorization and analysis are subject to coders’ interpretation. Future research should expand the participant pool and explore additional research domains, diverse geographic and institutional contexts, and varying CRP roles. Investigating how different institutional practices, guidelines, and cultures influence prescreening strategies could unravel more nuanced insights.

Additionally, while freelisting provides a structured mechanism to extract salient factors from participants’ responses, it may inadvertently omit subtle yet critical nuances and contexts that permeate CRPs’ experiences and decision-making. Future research should focus on the social determinants of health to ensure a more inclusive approach. Further, based on our findings, we plan to evaluate the effectiveness of CRPs’ prescreening practices.

Moreover, while the online survey offers a broad reach, it may have predisposed the findings to certain biases. Technological familiarity, digital literacy, and the absence of real-time clarification or probing might have shaped the responses, potentially skewing the results toward a more digitally comfortable and articulate demographic.

Finally, the intersection of technology and clinical research prescreening presents a fertile ground for exploration and innovation. Investigating the impacts and utilities of digital health tools, electronic health records (EHRs), artificial intelligence (AI), and predictive analytics in optimizing the prescreening process could pave the way for developing automated, efficient, and sophisticated eligibility determination strategies. For instance, integrating AI and predictive analytics with EHRs could automate the initial stages of eligibility screening, identifying potential participants based on predefined criteria and enabling CRPs to focus on more nuanced and context-specific prescreening considerations.

## Conclusion

This study offers a comprehensive and nuanced perspective on the practices and preferences of CRPs in the pivotal task of eligibility prescreening across diverse disease domains. Identifying universal and domain-specific elements lays the foundation for refining recruitment strategies, enhancing training programs, and developing tailored resources. The balance between generalized approaches and specialized nuances is crucial for fostering a clinical research environment that is ethical, efficient, and participant-centered. Through these insights, we contribute to the ongoing endeavor to advance clinical research practices to improve patient outcomes and medical advancements.

## Supporting information

Idnay et al. supplementary material Idnay et al. supplementary material

## Supplementary material

The supplementary material for this article can be found at https://doi.org/10.1017/cts.2024.617.
