# Supplementary material for: Clinical researchers’ insights on key data for eligibility screening in clinical studies
Source: J Clin Transl Sci. 2024 Oct 16;8(1):e167. doi: 10.1017/cts.2024.617 (PMC11604510; doi:10.1017/cts.2024.617)
Supplement: Idnay et al. supplementary material [file S2059866124006174sup001.docx]

Clinical Researchers' Insights on Key Data for Eligibility Screening in Clinical Studies

**SUPPLEMENTARY MATERIALS**

**Supplementary File A:** Freelisting Survey Instrument

In the following three questions, we will ask you to list down everything that comes to your mind in response to the question. Please note that there is no right or wrong answer. All that is important is that you indicate how you feel or think personally.

1. Please list all the information you look for in the **patient's medical records** to determine their potential eligibility for the clinical research you are recruiting for.

Please enter one information per answer field and click on "Add more" button to enter more information.

- 1. Free-text form field with “Add More” button if participant would like to list more.
  2. Optional: Please feel free to expand on your answers or share your thoughts on determining the eligibility of potential participants by looking for information in their medical records.

1. Please list all the information you **ask potential participants or their caregiver** to determine the potential participant's eligibility for the clinical research you are recruiting for.

   Please enter one information per answer field and click on "Add more" button to enter more information.
   1. Free-text form field with “Add More” button if participant would like to list more.
   2. Optional: Please feel free to expand on your answers or share your thoughts on determining the eligibility of potential participants by asking from information from the potential participant or their caregiver.
2. Please list all the information you **discuss with the principal investigator** to determine the potential participant's eligibility for the clinical research you are recruiting for.
   Please enter one information per answer field and click on "Add more" button to enter more information.
   1. Free-text form field with “Add More” button if participant would like to list more.
   2. Optional: Please feel free to expand on your answers or share your thoughts on determining the eligibility of potential participants by discussing certain patient information with the principal investigator.

**Supplementary Table S1.** Alphabetical Listing of Categories and Their Associated Synonyms During Potential Participant Inquiry.

| **NEUROLOGICAL DISORDERS** | |
| --- | --- |
| **Category (N=15)** | **Synonym (N= 60)** |
| Clinical care history | - Hospitalization history - Scheduled procedures |
| Clinical documentation | - Medical records access |
| Demographics | - Age - Demographics - Education - Ethnicity - Gender - Language - Race |
| Family history | - Family history |
| Functional status | - Activities of daily living - Motor function - Patient-reported outcome scores |
| History of present illness | - Cognitive status - Diagnosis - Diagnosis duration - Disease knowledge - Disease status - Mood - Neurological diagnosis - Neurological history - Neurological impairment - Onset age - Symptoms - Time of event |
| Past medical history | - Allergies - Comorbidities - Medical history - Pregnancy - Psychiatric history |
| Past surgical history | - Surgical history |
| Personal information | - Date of birth - Name - Residence location |
| Research Eligibility | - Capacity to consent - Caregiver availability - Magnetic resonance imaging contraindications - Protocol eligibility criteria - Research eligibility |
| Research participation | - Ability to attend study visits - Ability to participate - Ability to perform research procedures - Family's willingness in research participation - Interest in research participation - Participant concerns about research - Research participation history - Willingness to participate in research |
| Social history | - Criminal history - Living situation - Social support |
| Harmful substance use | - Alcohol use history - Smoking history - Substance use history |
| Treatment or medication | - Current treatment/intervention - Medication list - Past treatment/intervention - Prior medications |
| Visit data | - Height - Weight |
| **RARE DISEASES** | |
| **Category (N=14)** | **Synonym (N= 62)** |
| Clinical care history | - Clinical visit schedule - Healthcare provider - Hospitalization history |
| Clinical documentation | - Medical records access |
| Demographics | - Age - Gender - Language - Occupation |
| Diagnostic results | - Genetic testing result - Imaging results - Laboratory results - Tumor size |
| Family history | - Family history |
| History of present illness | - Clinical presentation - Cognitive Status - Diagnosis - Diagnosis age - Diagnosis date - Disease status - Overall health status - Symptom onset |
| Obstetric and gynecological history | - Birth control - Birth history - Fetal abnormalities - Gestational age - Last menstrual period - Pregnancy status - Pregnancy type |
| Past medical history | - Allergies - Behavior history - Cancer history - Claustrophobia - Comorbidities - Disability history - Medical history - Past injury - Pulmonary exacerbation - Raynaud’s syndrome - Sleep hours - Vaccination status |
| Past surgical history | - Surgical history - Transplant history |
| Personal information | - Date of birth - Name - Residence location |
| Physical function | - Functional status |
| Research eligibility | - Adherence - Capacity to consent - COVID symptoms - Flu symptoms |
| Research participation | - Ability to participate - Ability to perform research procedures - Availability to attend study visit - Caregiver availability - Interest in research participation - Participant concerns about research - Principal investigator’s availability - Protocol eligibility criteria - Research participation history - Willingness to participate in research |
| Harmful substance use | - Nicotine - Smoking history |
| **OTHER DISEASES** | |
| **Category (N=17)** | **Synonym (N=62)** |
| Clinical care history | - Clinical visit history - Healthcare provider - Institutionalization - Patient information - Treatment Site |
|  |  |
|  |  |
|  |  |
| Demographics | - Age - Biologic sex - Demographics - Ethnicity - Gender - Language |
|  |  |
|  |  |
| Diagnostic results | - Imaging results - Laboratory results |
|  |  |
| Family history | - Family history |
| History of present illness | - Co-morbidities - Diagnosis - Disease duration - Disease status - Sleep history - Symptom duration - Symptoms |
| Obstetric and gynecological history | - Last menstrual period - Pregnancy outcome - Pregnancy Risk - Pregnancy status - Pregnancy status - Pregnancy type |
|  |  |
| Other | - Currently on treatment authorization request - General demeanor - Past Self-Reported Data |
|  |  |
| Past medical history | - Allergies - COVID status - Neurological history - Past medical history - Past treatment/intervention - Vaccination status |
|  |  |
|  |  |
|  |  |
| Past surgical history | - Past surgical history - Transplant history |
| Personal information | - Contact information - Residence location |
| Physical activity status | - Activity level - Mobility |
| Research eligibility | - Adherence - Capacity to consent - Protocol eligibility criteria |
| Research participation | - Ability to participate - Ability to perform research procedures - Availability to attend study visit - Interest in research participation - Research participation history - Willingness to participate in research |
|  |  |
|  |  |
|  |  |
| Social History | - Incarceration - Living situation - Occupation |
| Harmful substance use | - Alcohol use history - Smoking history - Substance use history |
| Treatment or medication | - Medication list - Prior medications |
|  |  |
| Visit data | - Blood pressure - Height - Weight |
|  |  |

**Supplementary Table S2**. Alphabetical Listing of Categories and Their Associated Synonyms During Medical Records Review.

| **NEUROLOGICAL DISORDERS** | | | | |  |
| --- | --- | --- | --- | --- | --- |
| **Category (N=16)** | **Synonym (N=68)** | | | |  |
| Clinical care history | - Healthcare provider - Hospitalization history - Scheduled procedures | | | |  |
| Clinical documentation | - Clinical notes | | | |  |
| Cognitive test results | - Mini-mental Status Examination - Montreal Cognitive Assessment - Neuropsychological test results | | | |  |
| Demographics | - Age - Biologic sex - Demographics - Ethnicity - Gender - Language - Race | | | |  |
| Diagnostic results | - Genetic testing result - Laboratory results - Laboratory results: Cerebrospinal fluid - Imaging results: Magnetic resonance imaging - Imaging results: General - Imaging results: Electroencephalogram - Imaging results: Dopamine transporter Scan - Imaging results: Positron emission tomography scan - Pathology results | | | |  |
| Family history | - Family history | | | |  |
| Functional status | - Functional status - Motor function - Pain scores - Patient-reported outcomes scores | | | |  |
| History of present illness | - Cognitive status - Diagnosis - Diagnosis date - Disease duration - Disease status - Neurological diagnosis - Neurological history - Neurological impairment - Onset age - Overall health status - Symptoms | | | |  |
| None | - None | | | |  |
| Past medical history | - Allergies - Comorbidities - Fall history - Medical history - Neurological comorbidities - Psychiatric history - Surgical history | | | |  |
| Personal information | - Contact information - Date of birth - Medical record number - Name - Occupation - Residence location | | | |  |
| Research Eligibility | - Adherence - Caregiver availability - Magnetic resonance imaging contraindications - Protocol eligibility criteria - Research eligibility | | | |  |
| Research participation | - Family's willingness in research participation - Interest in research participation - Referral for research - Research participation history | | | |  |
| Social history | - Living situation - Social History | | | |  |
| Treatment/Procedures | - Current treatment/intervention - Medication list - Prior procedures | | | |  |
| Visit data | - Body mass index | | | |  |
| **RARE DISEASES** | | | | |  |
| **Category (N=18)** | **Synonym (N=62)** | | | |  |
| Clinical care history | - Clinical visit schedule - Healthcare provider - Hospitalization history | | | |  |
| Clinical documentation | - Medical notes - Progress notes | | | |  |
| Demographics | - Age - Biologic sex - Demographics - Gender - Language | | | |  |
| Diagnostic results | - Cardiac test - Genetic testing result - Imaging results - Laboratory results - Pulmonary function test - Tumor size | | | |  |
| Family history | - Family history | | | |  |
| History of present illness | - Chief complaint - Clinical presentation - Cognitive Status - Diagnosis - Diagnosis date - Diagnosis method - Diagnostic criteria - Disease duration - Disease status - Overall health status - Symptom onset | | | |  |
| Mortality | - Mortality | | | |  |
| Obstetric and gynecological history | - Birth report - Fetal abnormalities - Gestational age - Pregnancy status - Pregnancy type | | | |  |
| Past medical history | - Cognitive disability - Comorbidities - Complications - Lung infection - Medical history - Pulmonary exacerbation - Seizures | | | |  |
| Past surgical history | - Implant history - Surgical history - Transplant history | | | |  |
| Personal information | - Date of birth - Residence location | | | |  |
| Physical function | - Bulbar function - Functional Status | | | |  |
| Recruitment | - Referral for research | | | |  |
| Research eligibility | - Capacity to consent | | | |  |
| Research participation | - Protocol eligibility criteria | | | |  |
| Harmful substance use | - Smoking history - Substance use history | | | |  |
| Treatment or medication | - Airway therapy - Current treatment/intervention - Medication list - Past treatment/intervention - Radiotherapy | | | |  |
| Visit data | - Body mass index - Vision - Vital signs - Weight | | | |  |
| **OTHER DISEASES** | | | | | |
| **Category (N=17)** | | | **Synonym (N=61)** | | |
| Clinical care history | | - Admission status - Clinical visit history - Healthcare provider - Hospitalization - Institutionalization | |  |  |
| Clinical documentation | | - Clinical notes - Medical records access - Progress notes | |  |  |
| Demographics | | - Age - Biologic sex - Demographics - Ethnicity - Gender - Language - Race | |  |  |
| Diagnostic results | | - Genetic test results - Imaging results - Laboratory results - Pathology results | |  |  |
| Family history | | - Family history | |  |  |
| History of present illness | | - Diagnosis - Disease duration - Disease severity - Disease status - Life expectancy - Symptoms - Transplant eligibility | |  |  |
| Obstetric and gynecological history | | - Conception method - Pregnancy outcome - Pregnancy status - Pregnancy status - Pregnancy type | |  |  |
| Past medical history | | - Allergies - Co-morbidities - COVID status - Infection history - Newborn evaluation - Past medical history | |  |  |
| Past surgical history | | - Past surgical history - Transplant history | |  |  |
| Personal information | | - Contact information - Residence location | |  |  |
| Physical activity status | | - Activity level | |  |  |
| Research eligibility | | - Adherence - Mini-mental Status Examination - Protocol eligibility criteria | |  |  |
| Research participation | | - Availability to attend study visit - Research participation history - Willingness to participate in research | |  |  |
| Social History | | - Incarceration | |  |  |
| Harmful substance use | | - Smoking history - Substance use history | |  |  |
| Treatment or medication | | - Current treatment/intervention - Medication list - Past treatment/intervention - Prior medications | |  |  |
| Visit data | | - Blood pressure - Body mass index - Height - Vital signs - Weight | |  |  |

**Supplementary Table S3**. Alphabetical Listing of Categories and Their Associated Synonyms During Principal Investigator Inquiry.

| **NEUROLOGICAL DISEASES** | | | |
| --- | --- | --- | --- |
| **Category (N=17)** | | | **Synonym (N=70)** |
| Clinical care history | | | - Healthcare provider - Hospitalization history - Scheduled procedures |
| Clinical documentation | | | - Clinical notes |
| Cognitive test results | | | - Intelligence quotient - Neuropsychological test results |
| Demographics | | | - Age - Demographics - Ethnicity - Language |
| Diagnostic results | | | - Genetic testing result - Imaging results: Electroencephalogram - Imaging results: General - Imaging results: Magnetic resonance imaging - Imaging results: Positron emission tomography scan - Laboratory results: Cerebrospinal fluid - Laboratory results: General - Pathology results |
| Family history | | | - Family history |
| Functional status | | | - Activities of daily living - Functional status - Motor function - Risk assessment |
| History of present illness | | | - Cognitive status - Diagnosis - Diagnosis date - Diagnosis duration - Disease status - Mental status - Mood - Neurological diagnosis - Neurological history - Neurological impairment - Onset age - Overall health status - Prognosis - Symptoms - Time of event |
| None | | | - None |
| Past medical history | | | - Comorbidities - Medical history - Obesity history - Psychiatric history |
| Past surgical history | | | - Surgical history |
| Personal information | | | - Date of birth - Residence location |
| Research Eligibility | | | - Adherence - Capacity to consent - Magnetic resonance imaging contraindications - Positron emission tomography scan contraindications - Principal investigator's eligibility determination - Protocol eligibility criteria - Research eligibility |
| Research participation | | | - Ability to participate - Ability to perform research procedures - Interest in research participation - Participant concerns about research - Participant result access - Protocol information - Research participation history - Study logistics - Willingness to participate in research |
| Social history | | | - Social support |
| Treatment or medication | | | - Current treatment/intervention - Medication list - Past treatment/intervention - Prior medications |
| Visit data | | | - Blood pressure - Body mass index - Vital signs |
| **RARE DISEASES** | | | |
| **Category (N=14)** | **Synonym (N=53)** | | |
| Clinical care history |  | | |
| Demographics | - Age - Gender - Race | | |
| Diagnostic results | - Cardiac test - Genetic testing result - Laboratory results - Pulmonary function test - Tumor size | | |
| History of present illness | - Cognitive Status - Diagnosis - Diagnosis date - Diagnosis impact - Diagnosis method - Disease status - Symptom onset | | |
| Obstetric and gynecological history | - Birth control - Fetal abnormalities - Gestational age - Pregnancy status - Pregnancy type | | |
| Past medical history | - Behavior history - Cognitive disability - Comorbidities - Lung infection - Medical history - Psychiatric history - Pulmonary exacerbation | | |
| Past surgical history | - Transplant history | | |
| Personal information | - Residence location | | |
| Physical function | - Bulbar function - Functional Status | | |
| Research eligibility | - Adherence - Principal investigator’s eligibility determination | | |
| Research participation | - Ability to participate - Ability to perform research procedures - Availability to attend study visit - Caregiver availability - Family concerns - Interest in research participation - Participant concerns about research - Principal investigator’s availability - Protocol eligibility criteria - Willingness to participate in research | | |
| Harmful substance use | - Alcohol use history - Smoking history | | |
| Treatment or medication | - Airway therapy - Current treatment/intervention - Medication list - Past treatment/intervention - Prior medications | | |
| Visit data | - Physical exam - Vision | | |
| **OTHER DISEASES** | | | |
| **Category (N=16)** | | **Synonym (N=60)** | |
| Clinical care history | | - Admission status - Clinical visit history - Healthcare provider - Treatment Site | |
| Clinical documentation | | - Medical records access | |
| Demographics | | - Age - Biologic sex - Demographics - Gender - Language - Race | |
| Diagnostic results | | - Genetic test results - Imaging results - Laboratory results - Pathology results | |
| History of present illness | | - Chief complaint - Diagnosis - Disease duration - Disease severity - Disease status - Symptom duration - Symptoms | |
| Obstetric and gynecological history | | - Pregnancy outcome - Pregnancy status - Pregnancy type | |
| Past medical history | | - Allergies - Co-morbidities - COVID status - Past medical history - Past surgical history - Surgical candidacy - Transplant history | |
| Personal information | | - Residence location | |
| Physical activity status | | - Mobility | |
| Recruitment | | - Informed consent - Principal investigator’s approval to recruit - Research referral | |
| Research eligibility | | - Adherence - Capacity to consent - Dental screening - Glucose management - PI's eligibility determination - Protocol eligibility criteria - Risk screening | |
| Research participation | | - Ability to participate - Ability to perform research procedures - Availability to attend study visit - Research participation history - Willingness to participate in research | |
| Social History | | - Occupation | |
| Harmful substance use | | - Smoking history - Smoking status | |
| Treatment or medication | | - Current treatment/intervention - Medication list - Past treatment/intervention - Treatment/intervention history | |
| Visit data | | - Blood pressure - Height - Vital signs - Weight | |

**Supplementary Table S4.** Synonyms and salience score in neurological disorders domain.

|  | **Potential participant inquiry** | | | **Medical records review** | | | **Principal investigator inquiry** | | |
| --- | --- | --- | --- | --- | --- | --- | --- | --- | --- |
|  | Item | n | Salience score | Item | n | Salience score | Item | n | Salience score |
| 1 | Gender | 10 | 0.204 | Age | 34 | 0.690 | Age | 10 | 0.193 |
| 2 | Medication list | 18 | 0.194 | Diagnosis | 20 | 0.283 | Past medical history | 11 | 0.139 |
| 3 | Past medical history | 11 | 0.162 | Medication list | 19 | 0.180 | Medication list | 13 | 0.138 |
| 4 | Ability to attend study visits | 8 | 0.134 | Residence location | 13 | 0.152 | Diagnosis | 9 | 0.118 |
| 5 | Willingness to participate in research | 12 | 0.103 | Past medical history | 14 | 0.135 | Laboratory results | 6 | 0.099 |
| 6 | Diagnosis | 8 | 0.095 | Comorbidities | 9 | 0.103 | PI’s eligibility determination | 6 | 0.091 |
| 7 | Family history | 6 | 0.093 | Family history | 6 | 0.086 | Protocol eligibility criteria | 5 | 0.088 |
| 8 | Symptoms | 9 | 0.087 | Date of birth | 4 | 0.074 | Family history | 6 | 0.065 |
| 9 | Capacity to consent | 5 | 0.071 | Language | 7 | 0.073 | Imaging results | 6 | 0.061 |
| 10 | Participant concerns about research | 4 | 0.063 | Research participation history | 8 | 0.072 | Demographics | 3 | 0.061 |
| 11 | Residence location | 6 | 0.061 | Gender | 6 | 0.068 | Cognitive status | 4 | 0.055 |
| 12 | Date of birth | 4 | 0.060 | Neurological diagnosis | 4 | 0.061 | Residence location | 4 | 0.05 |
| 13 | Neurological history | 6 | 0.059 | Laboratory results | 5 | 0.060 | Symptoms | 6 | 0.049 |
| 14 | Surgical history | 7 | 0.059 | Symptoms | 8 | 0.060 | Disease status | 3 | 0.048 |
| 15 | Psychiatric history | 4 | 0.052 | Ethnicity | 6 | 0.057 | Psychiatric history | 6 | 0.045 |
| 16 | Ability to participate | 3 | 0.048 | Functional status | 4 | 0.056 | Neurological impairment | 3 | 0.043 |
| 17 | Onset age | 3 | 0.048 | Clinical notes | 4 | 0.054 | Protocol information | 4 | 0.041 |
| 18 | MRI contraindications | 3 | 0.046 | Cognitive status | 3 | 0.047 | Ability to perform research procedures | 2 | 0.041 |
| 19 | Interest in research participation | 3 | 0.043 | Psychiatric history | 4 | 0.047 | None | 2 | 0.041 |
| 20 | Comorbidities | 3 | 0.041 | Imaging results: MRI | 4 | 0.045 | Comorbidities | 2 | 0.041 |
| 21 | Patient-reported outcome scores | 2 | 0.041 | Imaging results | 3 | 0.044 | Capacity to consent | 2 | 0.041 |
| 22 | Neurological impairment | 4 | 0.039 | Hospitalization history | 4 | 0.042 | MRI contraindications | 3 | 0.039 |
| 23 | Ability to perform research procedures | 3 | 0.038 | Surgical history | 8 | 0.042 | Research eligibility | 3 | 0.037 |
| 24 | Protocol eligibility criteria | 2 | 0.037 | Allergies | 2 | 0.041 | Research participation history | 2 | 0.031 |
| 25 | Alcohol use history | 2 | 0.035 | Interest in research participation | 2 | 0.037 | Surgical history | 2 | 0.029 |
| 26 | Substance use history | 3 | 0.035 | Neurological history | 5 | 0.037 | Willingness to participate in research | 4 | 0.028 |
| 27 | Caregiver availability | 2 | 0.033 | Genetic testing result | 4 | 0.035 | Imaging results: MRI | 3 | 0.028 |
| 28 | Ethnicity | 2 | 0.033 | Body mass index | 2 | 0.031 | Study logistics | 2 | 0.028 |
| 29 | Neurological diagnosis | 3 | 0.033 | Disease status | 3 | 0.029 | Ethnicity | 2 | 0.026 |
| 30 | Demographics | 2 | 0.032 | Healthcare provider | 2 | 0.029 | Social support | 4 | 0.024 |
| 31 | Activities of daily living | 2 | 0.031 | Laboratory results: CSF | 2 | 0.028 | Interest in research participation | 2 | 0.024 |
| 32 | Cognitive status | 2 | 0.029 | Onset age | 2 | 0.026 | Ability to participate | 2 | 0.023 |
| 33 | Living situation | 2 | 0.029 | MMSE | 3 | 0.024 | Adherence | 2 | 0.022 |
| 34 | Allergies | 1 | 0.020 | Protocol eligibility criteria | 2 | 0.024 | Language | 2 | 0.021 |
| 35 | Criminal history | 1 | 0.020 | Race | 4 | 0.023 | Pet scan contraindications | 1 | 0.020 |
| 36 | Current treatment/intervention | 1 | 0.020 | Scheduled procedures | 1 | 0.02 | Motor function | 1 | 0.020 |
| 37 | Diagnosis duration | 1 | 0.020 | Disease duration | 1 | 0.018 | Functional status | 1 | 0.020 |
| 38 | Gender | 2 | 0.020 | Contact information | 1 | 0.017 | Activities of daily living | 1 | 0.020 |
| 39 | Scheduled procedures | 1 | 0.020 | Diagnosis date | 1 | 0.016 | Diagnosis date | 1 | 0.020 |
| 40 | Education | 1 | 0.018 | Research eligibility | 1 | 0.016 | Imaging results: pet scan | 1 | 0.020 |
| 41 | Family's willingness in research participation | 1 | 0.016 | Imaging results: DAT scan | 1 | 0.015 | Hospitalization history | 1 | 0.020 |
| 42 | Research participation history | 3 | 0.016 | Neurological comorbidities | 2 | 0.015 | Onset age | 1 | 0.020 |
| 43 | Disease knowledge | 1 | 0.015 | Neurological impairment | 1 | 0.015 | Mental status | 1 | 0.020 |
| 44 | Motor function | 1 | 0.015 | Biologic sex | 2 | 0.014 | Neurological diagnosis | 2 | 0.019 |
| 45 | Past treatment/intervention | 1 | 0.015 | Imaging results: EEG | 1 | 0.014 | Prior medications | 2 | 0.018 |
| 46 | Disease status | 2 | 0.014 | Living situation | 1 | 0.014 | Genetic testing result | 1 | 0.018 |
| 47 | Prior medications | 2 | 0.013 | Pain scores | 1 | 0.014 | Scheduled procedures | 2 | 0.017 |
| 48 | Height | 1 | 0.012 | Family's willingness in research participation | 1 | 0.012 | Current treatment/intervention | 2 | 0.017 |
| 49 | Research eligibility | 1 | 0.012 | MRI contraindications | 2 | 0.011 | Date of birth | 1 | 0.017 |
| 50 | Social support | 1 | 0.012 | Neuropsychological test results | 2 | 0.011 | Diagnosis duration | 1 | 0.016 |
| 51 | Race | 2 | 0.010 | Prior procedures | 2 | 0.010 | Intellectual quotient | 1 | 0.016 |
| 52 | Language | 1 | 0.008 | Adherence | 1 | 0.009 | Body mass index | 1 | 0.015 |
| 53 | Medical records access | 1 | 0.008 | Fall history | 1 | 0.008 | Imaging results: EEG | 1 | 0.015 |
| 54 | Name | 1 | 0.008 | Name | 1 | 0.008 | Past treatment/intervention | 1 | 0.015 |
| 55 | Pregnancy | 1 | 0.007 | Overall health status | 1 | 0.008 | Overall health status | 1 | 0.015 |
| 56 | Hospitalization history | 1 | 0.004 | Patient-reported outcome scores | 1 | 0.008 | Neurological history | 1 | 0.014 |
| 57 | Mood | 1 | 0.004 | Current treatment/intervention | 1 | 0.007 | Clinical notes | 1 | 0.012 |
| 58 | Smoking history | 1 | 0.004 | Demographics | 1 | 0.007 | Participant concerns about research | 1 | 0.010 |
| 59 | Time of event | 1 | 0.004 | MoCA | 2 | 0.007 | Participant result access | 1 | 0.010 |
| 60 | Weight | 1 | 0.002 | Pathology results | 1 | 0.007 | Mood | 1 | 0.008 |
| 61 | - | - | - | Referral for research | 1 | 0.006 | Risk assessment | 1 | 0.008 |
| 62 | - | - | - | Imaging results: pet scan | 1 | 0.005 | Blood pressure | 1 | 0.007 |
| 63 | - | - | - | MRN | 1 | 0.005 | Vital signs | 1 | 0.007 |
| 64 | - | - | - | None | 1 | 0.004 | Neuropsychological test results | 1 | 0.007 |
| 65 | - | - | - | Motor function | 1 | 0.003 | Obesity history | 1 | 0.005 |
| 66 | - | - | - | Social history | 1 | 0.003 | Pathology results | 1 | 0.005 |
| 67 | - | - | - | Occupation | 1 | 0.002 | Laboratory results: CSF | 1 | 0.004 |
| 68 | - | - | - | Caregiver availability | 1 | 0.001 | Time of event | 1 | 0.003 |
| 69 | - | - | - | - | - | - | Prognosis | 1 | 0.003 |
| 70 | - | - | - | - | - | - | Healthcare provider | 1 | 0.003 |

Note. MRI: magnetic resonance imaging; DAT: direct antiglobulin test; CSF: cerebrospinal fluid; MMSE: mini-mental status examination; MoCA: Montreal Cognitive Assessment; EEG: electroencephalogram; PI: principal investigator.

**Supplementary Table S5**. Synonyms and salience score in rare disease domain.

|  | **Potential participant inquiry** | | | **Medical records review** | | | **Principal investigator inquiry** | | |
| --- | --- | --- | --- | --- | --- | --- | --- | --- | --- |
|  | Item | n | Salience score | Item | n | Salience score | Item | n | Salience score |
| 1 | Availability to attend study visit | 12 | 0.205 | Age | 27 | 0.534 | Medication list | 12 | 0.192 |
| 2 | Age | 10 | 0.180 | Medication list | 16 | 0.177 | Medical history | 12 | 0.175 |
| 3 | Interest in research participation | 13 | 0.171 | Laboratory results | 17 | 0.157 | Age | 7 | 0.152 |
| 4 | Medication list | 13 | 0.170 | Genetic testing result | 12 | 0.156 | PI’s eligibility determination | 9 | 0.140 |
| 5 | Medical history | 12 | 0.110 | Diagnosis | 13 | 0.143 | Laboratory results | 7 | 0.100 |
| 6 | Fetal abnormalities | 4 | 0.083 | Imaging results | 7 | 0.101 | Diagnosis | 6 | 0.093 |
| 7 | Diagnosis | 5 | 0.071 | Fetal abnormalities | 6 | 0.099 | Protocol eligibility criteria | 5 | 0.083 |
| 8 | Ability to perform research procedures | 4 | 0.069 | Pulmonary function test | 8 | 0.086 | Pulmonary function test | 5 | 0.082 |
| 9 | Disease status | 5 | 0.066 | Date of birth | 4 | 0.080 | Disease status | 6 | 0.071 |
| 10 | Current treatment/intervention | 4 | 0.061 | Medical history | 8 | 0.077 | Genetic testing result | 4 | 0.071 |
| 11 | Capacity to consent | 4 | 0.056 | Comorbidities | 4 | 0.066 | Willingness to participate in research | 6 | 0.058 |
| 12 | Comorbidities | 3 | 0.055 | Disease status | 6 | 0.061 | Comorbidities | 3 | 0.057 |
| 13 | Research participation history | 6 | 0.054 | Residence location | 5 | 0.058 | Residence location | 5 | 0.053 |
| 14 | Laboratory results | 3 | 0.047 | Language | 5 | 0.051 | Adherence | 3 | 0.049 |
| 15 | Allergies | 2 | 0.042 | Functional status | 4 | 0.047 | Cognitive status | 4 | 0.045 |
| 16 | Airway therapy | 2 | 0.042 | Healthcare provider | 5 | 0.042 | Ability to participate | 3 | 0.045 |
| 17 | Medical records access | 3 | 0.040 | Clinical visit schedule | 2 | 0.040 | Availability to attend study visit | 2 | 0.038 |
| 18 | Behavior history | 2 | 0.039 | Surgical history | 7 | 0.037 | Functional status | 2 | 0.038 |
| 19 | Willingness to participate in research | 5 | 0.036 | Pulmonary exacerbation | 3 | 0.037 | Gestational age | 2 | 0.036 |
| 20 | Gestational age | 3 | 0.036 | Family history | 2 | 0.033 | PI's availability | 3 | 0.033 |
| 21 | Participant concerns about research | 2 | 0.036 | Medical notes | 2 | 0.033 | Fetal abnormalities | 2 | 0.033 |
| 22 | Diagnosis date | 2 | 0.035 | Pregancy status | 4 | 0.032 | Family concerns | 3 | 0.027 |
| 23 | Adherence | 2 | 0.035 | Cognitive status | 3 | 0.032 | Gender | 2 | 0.026 |
| 24 | COVID symptoms | 2 | 0.034 | Demographics | 2 | 0.032 | Current treatment/intervention | 2 | 0.024 |
| 25 | Genetic testing result | 3 | 0.032 | Progress notes | 4 | 0.031 | Smoking history | 2 | 0.023 |
| 26 | Protocol eligibility criteria | 2 | 0.031 | Gestational age | 2 | 0.030 | Tumor size | 1 | 0.022 |
| 27 | Date of birth | 2 | 0.031 | Gender | 3 | 0.026 | Participant concerns about research | 1 | 0.022 |
| 28 | Pregnancy status | 2 | 0.029 | Diagnosis date | 2 | 0.025 | Behavior history | 1 | 0.022 |
| 29 | Cancer history | 2 | 0.027 | Symptom onset | 3 | 0.023 | Caregiver availability | 1 | 0.022 |
| 30 | Birth control | 2 | 0.027 | Current treatment/intervention | 2 | 0.022 | Diagnosis date | 1 | 0.018 |
| 31 | Residence location | 2 | 0.026 | Pregnancy type | 2 | 0.020 | Birth control | 1 | 0.017 |
| 32 | Language | 2 | 0.025 | Birth report | 1 | 0.020 | Transplant history | 1 | 0.016 |
| 33 | Prior medications | 2 | 0.025 | Chief complaint | 1 | 0.020 | Lung infection | 2 | 0.015 |
| 34 | Past treatment/intervention | 2 | 0.024 | Body mass index | 1 | 0.020 | Prior medications | 1 | 0.014 |
| 35 | Healthcare provider | 2 | 0.021 | Weight | 1 | 0.018 | Physical examination | 1 | 0.014 |
| 36 | Ability to participate | 1 | 0.021 | Airway therapy | 2 | 0.017 | Psychiatric history | 1 | 0.014 |
| 37 | Symptom onset | 1 | 0.021 | Clinical presentation | 1 | 0.017 | Interest in research Participation | 1 | 0.013 |
| 38 | Diagnosis age | 1 | 0.021 | Complications | 1 | 0.017 | Pulmonary exacerbation | 1 | 0.013 |
| 39 | Clinical presentation | 1 | 0.021 | Tumor size | 1 | 0.017 | Ability to perform research procedures | 1 | 0.011 |
| 40 | Cognitive status | 1 | 0.021 | Substance use history | 1 | 0.016 | Symptom onset | 1 | 0.011 |
| 41 | Vaccination status | 2 | 0.019 | Past treatment/intervention | 2 | 0.015 | Diagnosis impact | 1 | 0.011 |
| 42 | Sleep hours | 1 | 0.019 | Mortality | 1 | 0.015 | Cognitive disability | 1 | 0.011 |
| 43 | Imaging results | 1 | 0.017 | Diagnostic criteria | 1 | 0.015 | Vision | 1 | 0.011 |
| 44 | Overall health status | 1 | 0.016 | Transplant history | 3 | 0.014 | Diagnosis method | 1 | 0.011 |
| 45 | Smoking history | 3 | 0.014 | Disease duration | 1 | 0.013 | Airway therapy | 1 | 0.009 |
| 46 | Flu symptoms | 1 | 0.014 | Overall health status | 1 | 0.013 | Healthcare provider | 1 | 0.007 |
| 47 | Birth history | 1 | 0.014 | Bulbar function | 1 | 0.013 | Cardiac test | 1 | 0.007 |
| 48 | Tumor size | 1 | 0.014 | Implant history | 1 | 0.013 | Pregnancy type | 1 | 0.007 |
| 49 | Steroid use | 3 | 0.013 | Cognitive disability | 1 | 0.010 | Bulbar function | 1 | 0.005 |
| 50 | Last menstrual period | 2 | 0.013 | Radiotherapy | 1 | 0.010 | Pregancy status | 1 | 0.005 |
| 51 | Pulmonary exacerbation | 2 | 0.013 | Hospitalization history | 1 | 0.010 | Alcohol use history | 1 | 0.004 |
| 52 | Name | 1 | 0.013 | Vision | 1 | 0.010 | Past treatment/intervention | 1 | 0.004 |
| 53 | Family history | 1 | 0.013 | Diagnosis method | 1 | 0.010 | Race | 1 | 0.004 |
| 54 | Hospitalization history | 1 | 0.012 | Protocol eligibility criteria | 2 | 0.009 |  |  |  |
| 55 | Vision | 1 | 0.010 | Vital signs | 2 | 0.008 |  |  |  |
| 56 | Occupation | 1 | 0.009 | Cardiac test | 1 | 0.008 |  |  |  |
| 57 | Disability history | 1 | 0.008 | Capacity to consent | 1 | 0.008 |  |  |  |
| 58 | Past injury | 1 | 0.007 | Smoking history | 2 | 0.007 |  |  |  |
| 59 | Surgical history | 2 | 0.006 | Lung infection | 1 | 0.007 |  |  |  |
| 60 | Transplant history | 1 | 0.006 | Referral for research | 1 | 0.004 |  |  |  |
| 61 | Gender | 1 | 0.005 | Seizures | 1 | 0.003 |  |  |  |
| 62 | Clinical visit schedule | 1 | 0.005 | Biologic sex | 1 | 0.003 |  |  |  |
| 63 | PI's availability | 1 | 0.005 | - | - | - |  |  |  |
| 64 | Nicotine | 1 | 0.005 | - | - | - |  |  |  |
| 65 | Raynauds syndrome | 1 | 0.005 | - | - | - |  |  |  |
| 66 | Pregnancy type | 1 | 0.005 | - | - | - |  |  |  |
| 67 | Caregiver availability | 1 | 0.004 | - | - | - |  |  |  |
| 68 | Functional status | 1 | 0.004 | - | - | - |  |  |  |
| 69 | Alcohol use history | 1 | 0.003 | - | - | - |  |  |  |
| 70 | Claustrophobia | 1 | 0.002 | - | - | - |  |  |  |

Note. MRI: magnetic resonance imaging; DAT: direct antiglobulin test; CSF: cerebrospinal fluid; MMSE: mini-mental status examination; MoCA: Montreal Cognitive Assessment; EEG: electroencephalogram; PI: principal investigator.

**Supplementary Table S6.** Synonyms and salience score in other diseases domain.

|  | **Potential participant inquiry** | | | **Medical records review** | | | **Principal investigator inquiry** | | |
| --- | --- | --- | --- | --- | --- | --- | --- | --- | --- |
|  | Item | n | Salience score | Item | n | Salience score | Item | n | Salience score |
| 1 | Age | 12 | 0.247 | Age | 39 | 0.763 | Age | 15 | 0.304 |
| 2 | Availability to attend study visit | 12 | 0.204 | Medication list | 20 | 0.230 | Medication list | 20 | 0.242 |
| 3 | Medication list | 15 | 0.197 | Laboratory results | 16 | 0.224 | Protocol eligibility criteria | 11 | 0.168 |
| 4 | Past medical history | 12 | 0.140 | Co-morbidities | 18 | 0.220 | Co-morbidities | 9 | 0.147 |
| 5 | Capacity to consent | 11 | 0.136 | Diagnosis | 9 | 0.132 | Past medical history | 7 | 0.111 |
| 6 | Ability to perform research procedures | 5 | 0.090 | Past medical history | 12 | 0.118 | Laboratory results | 9 | 0.110 |
| 7 | Co-morbidities | 7 | 0.087 | Imaging results | 8 | 0.078 | Adherence | 5 | 0.083 |
| 8 | Willingness to participate in research | 7 | 0.081 | Past surgical history | 9 | 0.072 | Imaging results | 6 | 0.082 |
| 9 | Interest in research participation | 4 | 0.065 | Gender | 5 | 0.068 | Diagnosis | 5 | 0.074 |
| 10 | Healthcare provider | 5 | 0.060 | Disease status | 6 | 0.067 | PI’s eligibility determination | 5 | 0.066 |
| 11 | Allergies | 3 | 0.057 | Biologic sex | 8 | 0.066 | Medical records access | 3 | 0.049 |
| 12 | Adherence | 3 | 0.051 | Residence location | 5 | 0.060 | Capacity to consent | 5 | 0.047 |
| 13 | Alcohol use history | 3 | 0.049 | Healthcare provider | 4 | 0.051 | Past treatment/intervention | 6 | 0.042 |
| 14 | Language | 4 | 0.046 | Language | 4 | 0.050 | Past surgical history | 5 | 0.042 |
| 15 | Ability to participate | 4 | 0.045 | Height | 3 | 0.048 | Ability to participate | 2 | 0.042 |
| 16 | Research participation history | 3 | 0.041 | Ethnicity | 3 | 0.040 | Disease status | 4 | 0.040 |
| 17 | Height | 3 | 0.040 | Demographics | 2 | 0.033 | Allergies | 2 | 0.039 |
| 18 | Laboratory results | 3 | 0.039 | Protocol eligibility criteria | 3 | 0.032 | Language | 2 | 0.035 |
| 19 | Family history | 2 | 0.036 | Race | 3 | 0.031 | Biologic sex | 3 | 0.032 |
| 20 | Residence location | 3 | 0.035 | Pregnancy status | 5 | 0.028 | Gender | 2 | 0.032 |
| 21 | Disease duration | 3 | 0.035 | Clinical notes | 3 | 0.027 | Availability to attend study visit | 2 | 0.031 |
| 22 | Demographics | 2 | 0.031 | Disease duration | 3 | 0.025 | Clinical visit history | 2 | 0.031 |
| 23 | Smoking history | 4 | 0.030 | Research participation history | 2 | 0.023 | Height | 2 | 0.030 |
| 24 | Pregnancy status | 3 | 0.030 | Pregnancy status | 2 | 0.023 | Blood pressure | 2 | 0.028 |
| 25 | Past treatment/intervention | 6 | 0.029 | Prior medications | 2 | 0.022 | PI’s approval to recruit | 2 | 0.028 |
| 26 | Covid status | 2 | 0.028 | Body mass index | 2 | 0.021 | Disease duration | 2 | 0.024 |
| 27 | Treatment site | 2 | 0.028 | Adherence | 2 | 0.020 | Residence location | 2 | 0.024 |
| 28 | Contact information | 2 | 0.027 | Substance use history | 1 | 0.020 | Willingness to participate in research | 2 | 0.024 |
| 29 | Past surgical history | 3 | 0.026 | Availability to attend study visit | 1 | 0.020 | Covid status | 2 | 0.021 |
| 30 | Living situation | 2 | 0.026 | Infection history | 2 | 0.019 | Chief complaint | 1 | 0.021 |
| 31 | Clinical visit history | 2 | 0.025 | Blood pressure | 2 | 0.018 | Pathology results | 1 | 0.021 |
| 32 | Substance use history | 2 | 0.024 | Activity level | 1 | 0.017 | Occupation | 1 | 0.018 |
| 33 | Gender | 2 | 0.023 | Family history | 1 | 0.017 | Glucose management | 1 | 0.017 |
| 34 | Patient information | 1 | 0.021 | Covid status | 1 | 0.016 | Research referral | 1 | 0.017 |
| 35 | Past self-reported data | 1 | 0.021 | Medical records access | 1 | 0.016 | Pregnancy outcome | 1 | 0.016 |
| 36 | Sleep history | 1 | 0.021 | Allergies | 1 | 0.016 | Demographics | 1 | 0.016 |
| 37 | Incarceration | 1 | 0.021 | Weight | 3 | 0.015 | Transplant history | 1 | 0.014 |
| 38 | Disease status | 1 | 0.021 | Vital signs | 3 | 0.014 | Disease severity | 1 | 0.014 |
| 39 | Symptoms | 3 | 0.020 | Genetic test results | 1 | 0.013 | Research participation history | 1 | 0.013 |
| 40 | Protocol eligibility criteria | 2 | 0.019 | Pregnancy type | 1 | 0.013 | Smoking status | 1 | 0.013 |
| 41 | Occupation | 1 | 0.018 | Life expectancy | 1 | 0.013 | Admission status | 2 | 0.011 |
| 42 | Pregnancy risk | 1 | 0.018 | Current treatment/intervention | 4 | 0.012 | Weight | 2 | 0.011 |
| 43 | Pregnancy type | 1 | 0.015 | Admission status | 2 | 0.012 | Symptom duration | 1 | 0.010 |
| 44 | Weight | 3 | 0.014 | Transplant history | 3 | 0.011 | Surgical candidacy | 1 | 0.010 |
| 45 | Activity level | 1 | 0.014 | Newborn evaluation | 1 | 0.011 | Genetic test results | 1 | 0.010 |
| 46 | Neurological history | 1 | 0.014 | Symptoms | 2 | 0.010 | Race | 1 | 0.010 |
| 47 | Transplant history | 1 | 0.014 | Conception method | 1 | 0.010 | Smoking history | 1 | 0.010 |
| 48 | Ethnicity | 1 | 0.013 | Progress notes | 1 | 0.009 | Treatment site | 1 | 0.010 |
| 49 | Imaging results | 1 | 0.013 | Incarceration | 1 | 0.009 | Treatment/intervention history | 1 | 0.010 |
| 50 | Pregnancy outcome | 1 | 0.013 | Contact information | 2 | 0.008 | Pregnancy status | 2 | 0.008 |
| 51 | Last menstrual period | 1 | 0.010 | MMSE | 1 | 0.008 | Risk screening | 1 | 0.008 |
| 52 | Pregnancy status | 1 | 0.010 | Pathology results | 1 | 0.008 | Dental screening | 1 | 0.007 |
| 53 | Prior medications | 1 | 0.009 | Clinical visit history | 1 | 0.007 | Pregnancy type | 1 | 0.007 |
| 54 | Diagnosis | 1 | 0.007 | Pregnancy outcome | 1 | 0.007 | Vital signs | 1 | 0.007 |
| 55 | Symptom duration | 1 | 0.007 | Disease severity | 1 | 0.007 | Ability to perform research procedures | 1 | 0.007 |
| 56 | Vaccination status | 1 | 0.007 | Transplant eligibility | 1 | 0.007 | Current treatment/intervention | 2 | 0.006 |
| 57 | General demeanor | 1 | 0.007 | Past treatment/intervention | 2 | 0.006 | Healthcare provider | 1 | 0.006 |
| 58 | Biologic sex | 1 | 0.005 | Hospitalization | 1 | 0.005 | Mobility | 1 | 0.003 |
| 59 | Institutionalization | 1 | 0.005 | Institutionalization | 1 | 0.004 | Symptoms | 1 | 0.003 |
| 60 | Blood pressure | 1 | 0.005 | Smoking history | 1 | 0.004 | Informed consent | 1 | 0.003 |
| 61 | Mobility | 1 | 0.004 | Willingness to participate in research | 1 | 0.004 | - | - | - |
| 62 | Currently on treatment authorized request | 1 | 0.003 | - | - | - | - | - | - |

Note. MRI: magnetic resonance imaging; DAT: direct antiglobulin test; CSF: cerebrospinal fluid; MMSE: mini-mental status examination; EEG: electroencephalogram; PI: principal investigator.
